# Structure-based identification of a new IAP-targeting compound that induces cancer cell death inducing NF-κB pathway

**DOI:** 10.1016/j.csbj.2021.11.034

**Published:** 2021-11-26

**Authors:** Federica Cossu, Simone Camelliti, Daniele Lecis, Luca Sorrentino, Maria Teresa Majorini, Mario Milani, Eloise Mastrangelo

**Affiliations:** a CNR-IBF, Consiglio Nazionale delle Ricerche – Istituto di Biofisica, Via Celoria, 26, I-20133 Milan, Italy; b Fondazione IRCCS Istituto Nazionale dei Tumori, Via Amadeo, 42, I-20133, Milano, Italy; c Dipartimento di Chimica, Università di Milano, Via Venezian, 21, I-20133 Milano, Italy

**Keywords:** IAP, inhibitor of apoptosis proteins, BIR, baculoviral IAP repeat, NF-kB, nuclear factor kappa-light-chain-enhancer of activated B cells, SM, Smac-mimetic, TNF, tumor necrosis factor, TRAF, TNF receptor associated factor, TGFβ, transforming growth factor-β, TAK1, (TGFβ)-activated kinase, TAB, TAK1 binding protein, Virtual screening, Inhibitor of apoptosis proteins, Baculoviral IAP repeat, IAP-antagonist, NF-kB, Drug discovery

## Abstract

Inhibitors of apoptosis proteins (IAPs) are validated onco-targets, as their overexpression correlates with cancer onset, progression, diffusion and chemoresistance. IAPs regulate cell death survival pathways, inflammation, and immunity. Targeting IAPs, by impairing their protein–protein interaction surfaces, can affect events occurring at different stages of cancer development.

To this purpose, we employed a rational virtual screening approach to identify compounds predicted to interfere with the assembly of pro-survival macromolecular complexes. One of the candidates, FC2, was shown to bind *in vitro* the BIR1 domains of both XIAP and cIAP2. Moreover, we demonstrated that FC2 can induce cancer cell death as a single agent and, more potently, in combination with the Smac-mimetic SM83 or with the cytokine TNF. FC2 determined a prolonged activation of the NF-κB pathway, accompanied to a stabilization of XIAP-TAB1 complex. This candidate molecule represents a valuable lead compound for the development of a new class of IAP-antagonists for cancer treatment.

## Highlights

• Virtual docking *vs* type I BIRs of IAPs identified *FC2* as a modulator of NF-kB.
• *FC2* is active as a single agent with no toxicity in normal cells.
• The cytotoxic activity of *FC2* is enhanced by TNF and by the Smac-mimetic SM83.
• *FC2* stabilizes XIAP/TAB1 interaction, prolonging the activation of NF-κB.

## Graphical abstract

**Figure:**
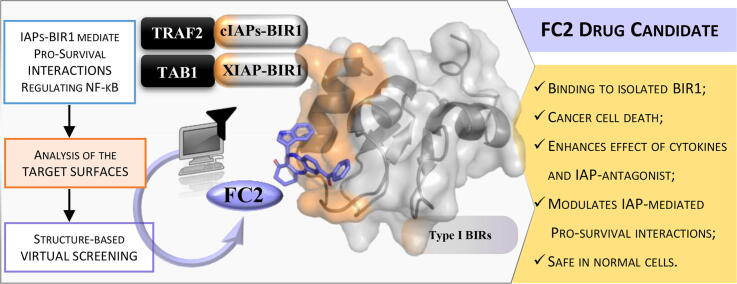

## 1 Introduction

Overexpression of Inhibitors of Apoptosis Proteins (IAPs) in cancer cells enhances their survival and resistance to anticancer agents. For this reason, type-II BIR domains of IAPs (*i.e.* BIR2 and BIR3 [1]) can be targeted with Smac-mimetics (SMs) compounds, some of which are now in advanced phases of clinical trials [2], [3], [4]. However, cases of resistance to SMs have been described in some cancer cell lines [5], due to NF-κB-mediated upregulation of cellular IAP2 (cIAP2).

Several evidences indicate that constitutive activation of NF-κB pathway and chronic inflammation play a major role in tumor development [6], [7]. IAP-mediated regulation of NF-κB signaling depends on different protein–protein (PP) complexes stabilized by type-I BIR domains (*i.e.* BIR1). More in detail, XIAP (X-chromosome-linked IAP) BIR1 domain forms a complex with transforming growth factor-β (TGFβ)-activated kinase (TAK1) binding protein, TAB1 [8], driving the NF-κB canonical pathway toward cell survival. In addition, cIAP1 and cIAP2 *i*) trigger the canonical NF-κB pathway toward cell survival, and *ii*) inhibit the non-canonical pathway hence blocking production of the death ligand TNF [9]. The cIAPs protein partners for these functions are TRAF1 (TNF receptor associated factor 1) and TRAF2 for the canonical pathway, and TRAF2 and TRAF3 for the non-canonical pathway.

Biochemical and crystallographic studies have shown that the TRAF1/2:cIAP1/2 interaction is based on the formation of the cIAP1:cIAP2 heterodimer [10], stabilized by the interaction of the RING domains, and the TRAF1:(TRAF2)_2_ heterotrimer. In any case, both cIAPs/TRAFs and XIAP/TAB1 interactions are mediated by the same surface of the respective BIR1 domains, which corresponds to the 2 N-ter helices of the domain (alpha1-alpha2). This remarkable common feature in XIAP- and cIAP2-BIR1 domains can be exploited to identify new classes of wide spectrum molecules for the modulation of the BIR1-mediated interactions. In this context, we already characterized the compound NF023, resulting from virtual screening of the LOPAC® library [11], [12], [13]. This candidate was found to disrupt cIAP2-BIR1/TRAF2 assembly and to interfere with the dimerization of XIAP-BIR1, a crucial event for activation of NF-κB. However, due to its chemical properties, its pharmacological development would require complex strategies to ensure optimal administration [14], [15].

In this paper, we report the identification, through *in silico* docking, of new BIR1 binders to be developed as cell death-inducing compounds. Our data show that one of the selected compounds, FC2, is able to reduce viability of different cancer cell lines in a dose dependent manner. Such an effect is more pronounced when FC2 is administered in combination with TNF or with the Smac-mimetic SM83 (namely 9a) [16], [17] in two out of three cancer cell lines tested. The treatment with FC2 of HEK293 cells overexpressing XIAP, promotes a prolonged activation of NF-κB along with the stabilization of XIAP-TAB1 interaction. Because of the multiplicity of roles of IAPs as “molecular switches”, FC2 can represent a starting tool to explore new pharmacological approaches that do not simply turn on/off a cellular pathway, but are rather able to tune it.

## 2 Material and methods

### 2.1 In silico docking analysis

In silico docking analysis targeting the BIR1 domains involved in the interactions between XIAP/ TAB1 and cIAP2/TRAF2 were performed to find compounds able to perturb the assembly of both complexes. The ChemBridge library (www.hit2lead.com) was used to screen 30 k low molecular weight compounds. First, the structural model of the domains was prepared (PDB code 3M1A, chain A for cIAP1-BIR1 [18], 3M0A, chain D for cIAP2-BIR1 [10], PDB code 2POP, chain B for XIAP-BIR1 [8]). Then, a grid delineating the protein region for the docking analysis was defined using a Grid Parameter File, GPF (cIAP1- and cIAP2-BIR1 grid centered near Met50/Met33, size: 28.5x28.5x22.5 Å^3^_,_ spacing 0.375 Å; XIAP-BIR1 grid centered near Phe1052, size: 47.3x47.3x47.3 Å^3^, spacing 0.375 Å). The GPFs were then used as input by AutoGrid4 (AutoDock4 package) to calculate the interaction energy of different kinds of atoms on the grid points. The ChemBridge library was treated with MolConvert (ChemAxon) to generate sdf format files with 3D coordinates for each compound, removing salts or additives. The program Babel converted all sdf into pdb files. After the addition of hydrogen atoms, Gasteiger charges, and the definition of the rotational constraints on every molecule in the library (MGLTools package: http://mgtools.scripps.edu/-), a DPF (Docking Parameter File) was generated for each compound to perform fifty independent genetic algorithm runs (GA). A first docking run was performed with AutoDock Vina package (vina.scripps.edu/) [19]. Then, the best ranked compounds (∼1%) were subjected to another virtual docking analysis with AutoDock4 [20]. The best ligands were selected according to their energy of interaction and to their water solubility (log SW > –4). Based on the location of the predicted binding sites on the XIAP-BIR1 surface, ten compounds (FC1–FC10) were selected as likely able to impair XIAP-BIR1 dimerization, whereas compounds FC11–FC20 were predicted to bind XIAP-BIR1 surface involved in the interaction with TAB1.

### 2.2 Chemicals and reagents

All compounds were purchased from Molport, Inc. as distributor of Chembridge Corp. First ten entries of Table S1, result from virtual docking targeting the surface of XIAP-BIR1 involved in homo-dimerization (FC1 to FC7, Chembridge IDs 7509055, 6409326, 5978186, 7630374, 7659647, 7463435, 7356505). Structural analogs of FC1, 4 and 6 with improved predicted solubility in water (FC1_1, FC4_1 and FC6_1) were also purchased (ChemBridge ID 6955650, 5216161 and 9288906, respectively). Compounds FC_8 to FC_17 (Chembridge IDs 7364714, 6044923, 7952747, 5722562, 7520752, 7994504, 7356135, 6721939, 5872899, 6122637) resulted from docking versus the surface of XIAP-BIR1 involved in the interaction with TAB1. FC2 structural analogs available from Chembridge Corp. (www.hittolead.com) were purchased to explore FC2 chemical modifications and investigate their ability to induce cell death. Chembridge IDs of FC2 structural analogs: (entry 1) 5878396, (entry 2, FC2) 6409326, (entry 3, FC3) 5978186, (entry 4) 6244152, (entry 5) 6433517, (entry 6) 6065770, (entry 7) 6968700, (entry 8) 7358852, (entry 9, FC9) 6044923, (entry 10) 6081028. FC2 NMR data from Chembridge Corp are reported in Fig. S5. Lipofectamine 2000 and Lipofectamine RNAiMAX (Thermo Fisher Scientific) reagents were used for plasmid and siRNA transfections, respectively.

### 2.3 Cell culturing

The human breast adenocarcinoma cell lines MDA-MB231, BT549, MDA-MB-468, HCC1419 and the ovarian SK-OV3 (from American Type Culture Collection) were cultured *in vitro* with RPMI-1640 medium (Lonza Group, Basel, CH) supplemented with 10% fetal bovine serum (FBS; EuroClone, Milan, IT), 2 mM glutamine, sodium pyruvate, and non-essential amino acids (Lonza Group). HEK293FT (Thermo Fisher Scientific) highly transfectable cell lines were cultured in DMEM with 10% FBS. The human foreskin BJ cells (from American Type Culture Collection) were cultured in E-MEM medium (Lonza Group) supplemented with 10% fetal bovine serum (FBS; EuroClone). All cells were grown at 37 °C in a fully humidified atmosphere with 5% CO_2_.

### 2.4 Cell viability assays

Cell viability assays were prepared by seeding MDA-MB231, BT-549, SK-OV3 and BJ cells (10^4^ cells/well) in 96 well plates. Cells were treated with the compounds from 1 µM to 100 µM in the absence/presence of TNF at the concentration of 50 ng/ml, or SM83 at the concentration of 1 to 100 nM. Infliximab (IFX) was administered ad 10 μg/mL. In order to determine the synergistic/additive effect of FC2 combination with TNF/SM83, MDA-MB-231 were treated with serial dilutions of FC2 (from 100 μM to 3.12 μM) and of TNF (500, 100, 20, 4, 0.8 ng/ml) or SM83 (100, 33.3, 11.1, 3.7, 1.2 nM). After 48 h, cell viability was evaluated by measuring cellular ATP content with CellTiter-Glo Luminescent Cell Viability Assay (Promega, Milan, Italy) according to manufacturer's protocol. Luminescence was measured using the multimode detection platform Spark 10 M (Tecan). IC50 values were calculated with Graph Pad. Synergy/additive effects were calculated with Synergy Finder [21]. Detection of caspases 3/7 activation was performed using CellEvent™ Caspase−3/7 Green Detection Reagent (Thermo Fisher Scientific) at a final concentration of 2 μM/well. Light microscope/fluorescence images were recorded at 48 h from treatments.

### 2.5 Expression and purification of cIAP2-BIR1 and XIAP-BIR1

The cIAP2-BIR1 and XIAP-BIR1 domains were expressed and purified as already described [11], [12], [13]. In brief, plasmids for cIAP2-BIR1 or XIAP-BIR1 expression were used to transform *Escherichia coli* strain BL21-CodonPlus (DE3)-RP competent cells (Agilent®). After induction with 0.5 mM isopropyl-β-D-thiogalactopyranoside (IPTG, Sigma-Aldrich), bacteria were grown for 3 h at 37 °C. After cell lysis, the expressed proteins were purified using Ni-NTA affinity column (His-trap FFcrude, GE Healthcare) and size exclusion chromatography (Superdex 75, GE Healthcare). Proteins were concentrated with Amicon Ultra centrifugal filters (with 3 kDa cut-off) in buffer 50 mM Tris-HCl pH 8.0, 200 mM NaCl, 10 mM DTT.

### 2.6 Thermal stability assay

Thermal denaturation assays were conducted in a MiniOpticon Real Time PCR Detection System (Bio-Rad), as already described [22] with protein/compounds concentrations 0.04/1 mM. Samples were heated from 20 to 95 °C (0.2 °C/min) measuring fluorescence intensity at the Sypro orange excitation/emission ranges 470–505/540–700 nm, respectively.

### 2.7 Trp fluorescence assays

Trp fluorescence variation was used to determine the protein–ligand dissociation constant K_d_. The fluorescence intensity emitted by XIAP-BIR1 Trp73 or cIAP2-BIR1 Trp76 were measured varying the concentration of the different ligands. The tests were performed with a spectral fluorimeter (Varian Cary Eclipse Fluorescence Spectrophotometer, Agilent Technologies) at 10 °C, recording the fluorescence emission spectra between 300 and 400 nm (excitation 280 nm). XIAP-/cIAP2-BIR1 protein samples were concentrated at 5/35 μM, respectively.

Two-fold dilutions of FC2 were prepared, to reach final concentrations ranging from 106 to 0.2 μM. 8 μl of each FC2 dilution were mixed with 180 μl of the protein solutions and fluorescence was measured in a 200 μl quartz cuvette. The K_d_ values were obtained through the GraFit5 program (Erithacus Software Limited, 2010), fitting the fluorescence values (F) with the following equation dependent on three parameters (M, m, K_d_):

$$\left[PI\right]=\frac{\left[P_{T}\right]+\left[I_{T}\right]+K_{d}-\sqrt{{\left(\left[P_{T}\right]+\left[I_{T}\right]+K_{d}\right)}^{2}-4\left[P_{T}\right]{\left[I_{T}\right]}^{2}}}{2}$$

$$F=M-\frac{\left(M-m\right)}{\left[P_{T}\right]}\left[\mathrm{PI}\right]$$

where [P_T_] and [I_T_] represent respectively the total concentration of protein and ligand, *M* and *m* indicate the maximum and minimum recorded fluorescence intensity value while [PI] expresses the amount of protein bound to the inhibitor.

### 2.8 Proteins detection in MDA-MB-231 lysates, cytoplasmic and nuclear extracts

MDA-MB-231 cells were pre-treated with 20 μM or 100 μM FC2 for 1 h. Subsequent stimulation with TNF was performed at a concentration of 50 ng/mL. After cells harvesting, cleared supernatants of cells lysates or cytoplasmic/nuclear extracts (obtained with NE-PER™ Nuclear and Cytoplasmic Extraction kit by Thermo Fisher Scientific) were separated by SDS-PAGE on precast 4–12% Bis-Tris NuPAGE gels (Thermo Fisher Scientific) and proteins were transferred to PVDF membranes (Merck Millipore, Darmstadt, Germany) using the XCell II blot module (Thermo Fisher Scientific). Membranes were then saturated for 60 min in Tris-buffered saline containing 4% BSA and incubated overnight with the anti-human primary antibodies recognizing cIAP1 #ab108361, phospho-NF-kB-p65 #3033, Myc-Tag #2278; Phospho-MKK3/MKK6 #9236, Phospho-TAK1 #4508 (Cell Signaling) cIAP2, p65 and Vinculin #V9131 (Sigma-Aldrich). After incubation with the proper secondary antibody (anti-rabbit and anti-mouse IgG were purchased from GE Healthcare, UK; all secondary antibodies were diluted 1:2000), proteins were detected by chemiluminescence (EuroClone).

### 2.9 XIAP over-expression by plasmid transfections

HEK293FT cells were transfected with pcDNA3-myc-XIAP (kindly provided by Kashkar, [23]) to over-express XIAP and with pCDNA3-GFP as control. HEK293FT cells were seeded in 10 cm Petri dishes to reach 80–90% confluence ON. The day after, cells were transfected with Lipofectamine 2000 (Thermo Fisher Scientific) according to manufacturer’s protocol. Briefly, both DNA and Lipofectamine 2000 were resuspended in Opti-MEM and incubated for 5 min. Then, the two mixtures were combined (DNA:Lipofectamine ratio = 1:3) to allow the formation of liposomes-DNA complexes for 30 min and then added to cells. Experiments were performed 48 h after transfection.

### 2.10 Immunoprecipitations to evaluate XIAP/TAB1 interaction with and without FC2 treatment

After 48 h from Myc-tagged XIAP or Myc-tagged eGFP plasmids transfection HEK293-FT cells were pre-treated with FC2 at a final concentration of 20 µM. A parallel set of cells was left untreated as control. After 30 min, cells were treated with TNF-α (50 ng/ml). After further 30 min, cells were harvested, washed with ice-cold PBS and lysed in E1A lysis buffer (ELB; 150 mM NaCl, 50 mM Hepes, pH 7.5, 5 mM EDTA, 0.5% NP40) supplemented with the mix of protease inhibitors at 4 °C for 30 min. Samples were clarified at 4 °C by centrifugation for 10 min at 13,000 rpm and then quantified with Bradford Assay. 1 mg of total protein extracts were incubated overnight at 4 °C with Protein-A-Sepharose (Sigma-Aldrich) and anti-Myc-Tag antibody #2278 (dilution 1:200, Cell Signaling Technology, Danvers, MA, USA). The Protein-A-Sepharose was previously washed with ELB buffer three times. The bound proteins and 50 µg of each lysate were analyzed by SDS-PAGE and immunoblotting with anti-human primary antibodies recognizing: XIAP #610763 (BD Biosciences, Franklin Lakes, New Jersey, USA), TAB1 #3226, TAK1 #5206 (Cell Signaling Technology, Danvers, MA, USA) and Vinculin #V9131 (Sigma-Aldrich).

### 2.11 Statistical analysis

All cell-based experiments were repeated in three independent experiments performed in technical triplicates. Results are presented as mean ± S.D, analyzed with GraphPad 9.0.2 (San Diego, CA, USA).

## 3 Results and discussion

### 3.1 Selection of FC2 as inducer of cancer cell death

#### 3.1.1 In silico docking

We performed *in silico* docking searches targeting the BIR1 domain surfaces involved in cIAP2/TRAF2 (chain D, PDB code 3M0A [10]), XIAP/TAB1 and XIAP/XIAP (chain B, PDB code 2POP [8]) interaction as previously described [11], [12], with the AutoDock VINA package (vina.scripps.edu/) [19], using the Chembridge library. For each protein target surface, the best 100 ranked compounds were analyzed more accurately using the AutoDock4 suite (autodock.scripps.edu/).

Among the compounds resulting from the docking versus XIAP-BIR1 homodimerization surface, the best 7 ranked hits were selected (including FC1 to FC7), and additional 3 structural analogs of FC1, 4 and 6 (FC1_1, FC4_1, FC6_1, respectively), with improved water solubility (LogSW > −4), were also included in the list. Further 10 compounds were chosen among the best hits from the docking results targeting the XIAP-BIR1/TAB1 structure (FC8–FC17). Notably, FC2 was found as able to target XIAP-BIR1 surface engaged in the interaction with TAB1, with comparable energies.

Interestingly, FC1 to 4, FC6 and FC9 were also found among the best ranked compounds resulting from the docking targeting cIAP2-BIR1.

Overall, a set of 20 compounds targeting XIAP- and cIAP2-BIR1 were selected for cell viability assays on MDA-MB-231 cell lines (Table S1).

#### 3.1.2 Cell toxicity assays and targeted *in silico* studies: selection of FC2

The selected compounds (Table S1) were analyzed in cell-based assays, to verify their toxicity on human breast adenocarcinoma cell line MDA-MB-231. Since TNF stimulates the TNF-R and the recruitment of all molecular actors (*i.e.* IAPs) triggering NF-κB pathway, the effect of each compound on cell viability was measured both as single agent (at 100, 10 and 1 μM) and in combination with TNF (50 ng/ml). Six compounds (FC2, FC3, FC4_1, FC7, FC11, FC14) showed appreciable toxicity at the highest concentration tested (100 µM) and other six were more active in combination with TNF (FC2, FC4, FC5, FC7, FC9, FC12). Only FC2 (chemical name 7-benzoyl-11-(1H-indol-3-yl)-2,3,4,5,10,11-hexahydro-1H-dibenzo[b,e][1], [4]diazepin-1-one, Table S1) showed toxicity also at lower concentrations (10 µM), enhanced in the presence of TNF. Moreover, FC2 did not show toxicity in BJ cell line (human primary fibroblast), thus providing encouraging data on its tolerability in normal tissue (Fig. S1, A).

**Fig. 1:**
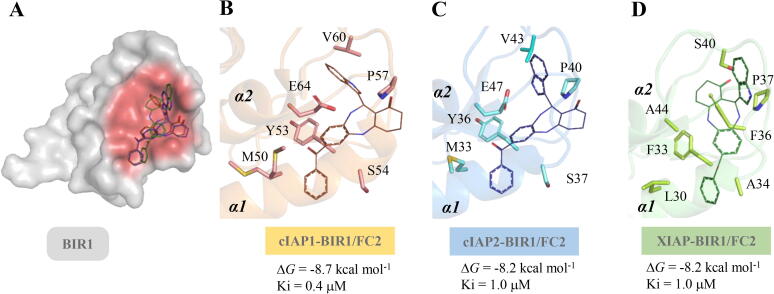
Comparison between FC2 poses in virtual docking versus cIAP1-, cIAP2- and XIAP-BIR1 α1-α2 structural hotspots. A) The BIR1 domain of homologous IAPs shown as grey surface. The structural hotspots involved in PPI with the partner proteins (TRAF2 or TAB1), the α1-α2 region of BIR1, are shown as red surface. FC2 (lines) predicted poses on cIAP1- (B, orange cartoon), cIAP2- (C, light blue cartoon) and XIAP-BIR1 (D, green cartoon) were superimposable, showing comparable free energies of binding (ΔG for cIAP2-BIR1/XIAP-BIR1 = −8.2 kcal/mol; ΔG for cIAP1-BIR1 = −8.7 kcal/mol). In sticks are highlighted the residues of α1-α2 region involved in the predicted interaction with FC2. Images were drawn with PyMOL (The PyMOL Molecular Graphics System, Version 2.0 Schrödinger, LLC.) (For interpretation of the references to colour in this figure legend, the reader is referred to the web version of this article.)

As described above, FC2 was found as ligand of XIAP-BIR1/TAB1 and of cIAP2-BIR1/TRAF2. No structures of other homologous human BIR1 domains in complex with partner proteins are deposited in the Protein Data Bank. However, Mace et al. mapped the interaction between cIAP1-BIR1 and TRAF2 [18]. In this work, the residues of cIAP1-BIR1 interacting *in vitro* with TRAF2 outline the same hotspot region of cIAP2-BIR1 shown to interact with TRAF2 in the crystal structure by Zheng et al. [10]. For this reason, we performed virtual docking of FC2 versus cIAP1-BIR1, in order to verify the ability of FC2 to target the homologous structural hotspot crucial for pro-survival protein–protein interactions. Fig. 1 summarizes the binding poses and energy values of FC2 to cIAP1-, cIAP2- and XIAP-BIR1 predicted in virtual docking. BIR1 residues interacting with FC2 are conserved among homologous domains (Fig. 1, B-C) and include some of the crucial residues identified as interactors of TRAF2 (M50/M33, E64/E47 of cIAP1/cIAP2) and TAB1 (see also Fig. S4).

#### 3.1.3 SAR analysis: FC2 chemical space exploration

**Fig. 2:**
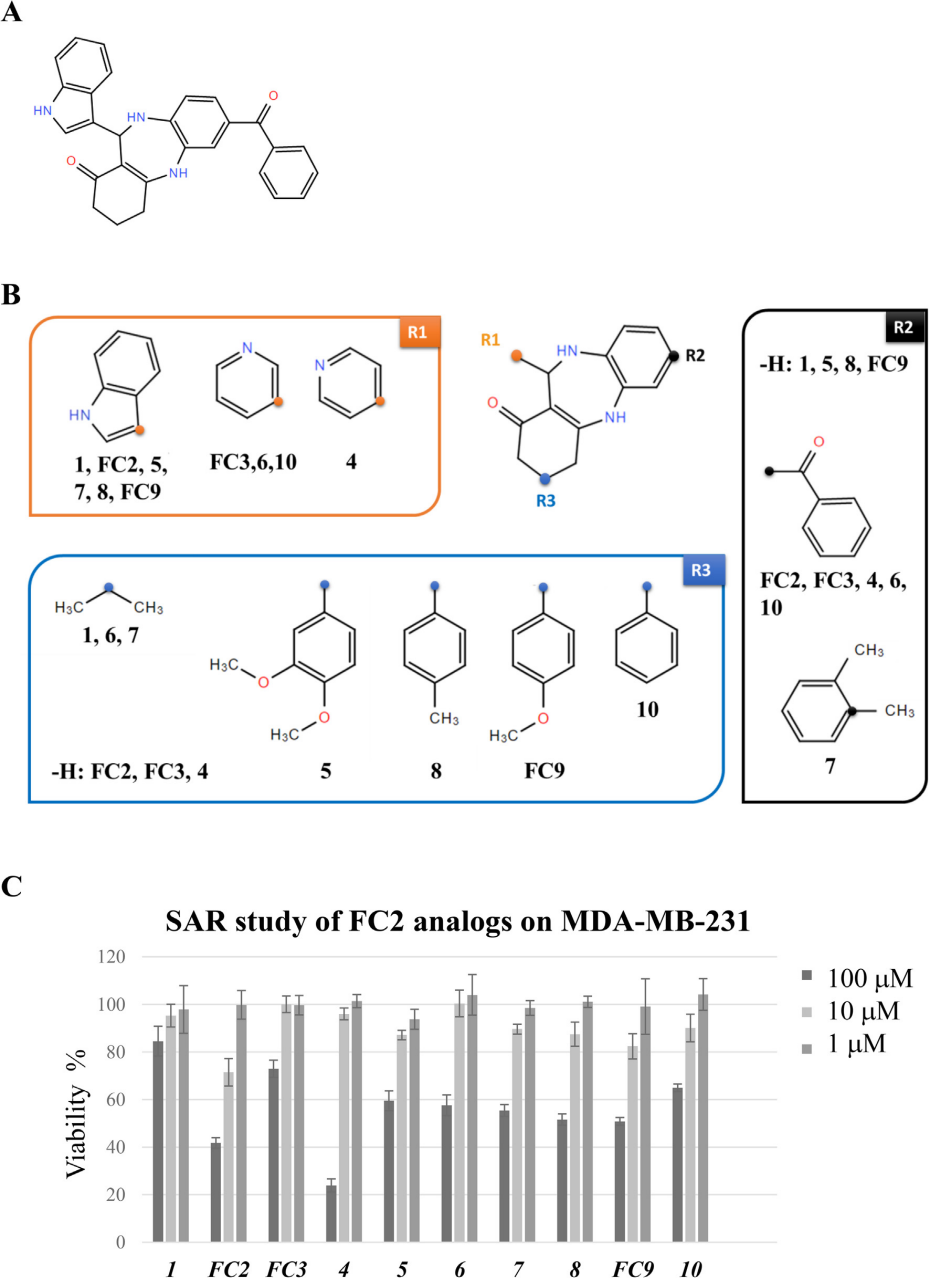
SAR study with FC2 analogs in cell viability assays. A) Chemical structure of FC2. B) Chemical structure of nine FC2 analogs available in the database (https://www.hit2lead.com/). The FC2-analogs have different chemical substitutions in R1, R2 and R3. C) Viability of MDA-MB-231 cells treated with FC2 and FC2-analogs at 3 different concentrations (1, 10, 100 μM). Among the FC2-analog compounds tested, FC2 shows the highest cytotoxicity at intermediate concentrations (10 μM, light grey bar). Significant cytotoxicity is observed for analog 4 at the highest concentration (100 μM, black bar). Mean values ± Standard deviation are shown.

In order to explore the chemical moieties essential/dispensable for FC2 cytotoxic activity, 9 structural analogs were selected from the ChemBridge library in which R1, R2 and R3 were substituted as shown in Fig. 2. Two analogs (FC3 and FC9) were already present in the first set of compounds selected by virtual docking (see 3.1.1). The other 7 analogues have been included in a further virtual docking analysis, performed as for the previous compounds, against cIAP2- and XIAP-BIR1. Among its structural analogs, FC2 displayed the best binding energies (Table S2). In cell-based assays, only FC2 and, with lower efficacy, analog 4 were able to reduce the viability of MDA-MB-231 cells. Analog 4 showed a significant toxicity at the highest concentration (100 μM), enhanced in combination with TNF or with the Smac-mimetic SM83 (data not shown).

Such preliminary SAR analysis suggests that any change in R2 or the addition of bulky moieties in R3 compromise the pro-death activity of FC2. As for R1, the indole substitution by a piperidine with nitrogen in para (analog 4) slightly reduces the activity, that is completely lost when the piperidine’s nitrogen is in meta (analog 5). Thus, the substituents in position R1 and R2, and the absence of substituents in position R3, appear to be essential for the activity of this class of compounds.

### 3.2 FC2 protein binding assays

The affinity of FC2 for the purified BIR1 domains was measured monitoring the fluorescence variation of the Trp residues upon addition of the drug candidate. FC2 displayed dissociation constants in the micromolar range for both the target domains *in vitro* (K_d_[cIAP2-BIR1] = 0.5 ± 0.2 µM; K_d_ [XIAP-BIR1] = 5.2 ± 1.0 µM). Differently from the cIAP2- and XIAP-BIR1 ligand NF023 [11], FC2 did not impair the dimerization of XIAP-BIR1, as demonstrated by SEC analysis (Table S3). Thermofluorimetric assays showed that FC2 induced a slight thermal destabilization of both cIAP2- and XIAP-BIR1 domains, decreasing their melting temperatures (T_M_) by 1–3 °C (Table S2).

### 3.3 Investigations of FC2 mechanism of action

#### 3.3.1 FC2 induces cell death as a single agent and FC2 toxicity is enhanced by TNF and SM

**Fig. 3:**
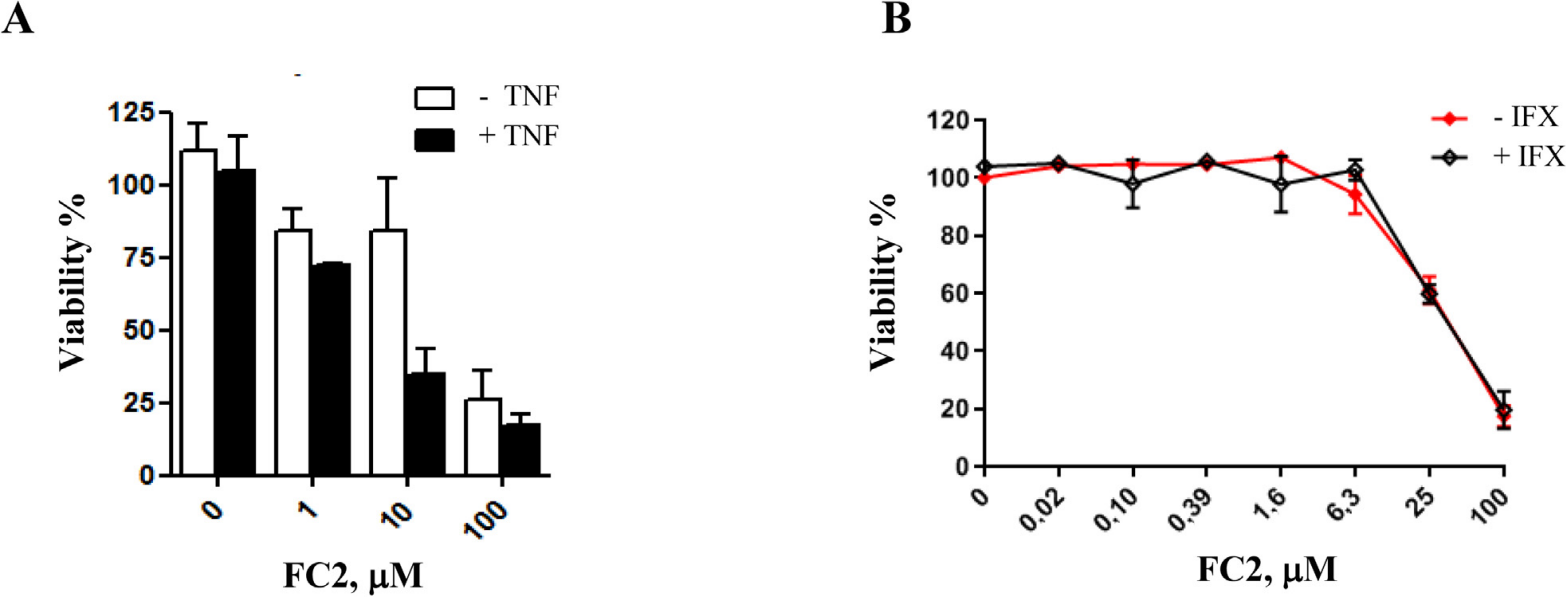
FC2 activity as a single agent and in combination with TNF. (A) Viability of MDA-MB-231 cells treated with FC2 alone (white bars) and in combination with recombinant TNF (50 ng/ml; black bars). (B) TNF-independent cytotoxicity of the FC2: MDA-MB-231 cell treated with different amounts of FC2 (from 0 to 100 μM) in the absence (red line) / presence (black line) of IFX (which blocks autocrine TNF). All the experiments were repeated both in technical (three for each experiment) and biological (three independent experiments) triplicate. Mean values ± Standard deviation are shown. Statistical analysis performed with GraphPad. (For interpretation of the references to colour in this figure legend, the reader is referred to the web version of this article.)

Since the targets of our study are PPIs regulating the NF-κB pathway, we assayed the pro-death activity of FC2 in combination with recombinant TNF to trigger NF-κB response. In MDA-MB-231 FC2 cytotoxicity was indeed enhanced by the addition of 50 ng/ml recombinant TNF (non-toxic as single agent) (Fig. 3, A) as shown by the variation of IC50 from 39.33 μM to 4.37 μM.

Moreover, FC2 reduces viability on both human breast adenocarcinoma cell line (BT549) and ovarian tumor cells (SK-OV3) (Fig. S2, A), with TNF enhanced effect in the latter (Fig. S2, B). Two additional cancer cell lines MDA-MB-468 (triple negative breast adenocarcinoma) and HCC1419 (HER2 + breast adenocarcinoma) were included in the study of FC2 toxicity. In MDA-MB-468 we observed similar toxicity effects compared to MDA-MB-231, while in HCC1419 the toxicity effect of FC2 was comparable to the effect observed in BT-549 (Fig. S1, B).

FC2 activity on MDA-MB-231, was retained also after blocking autocrine TNF with the antibody infliximab (IFX) (Fig. 3, B), thus demonstrating a TNF- independent cytotoxicity of the compound. This evidence shows that FC2 kills cancer cells through a different mechanism of action compared to SM-induced apoptosis [24].

**Fig. 4:**
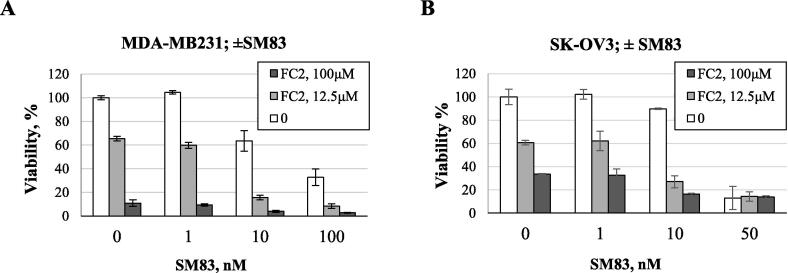
Enhanced FC2 cytotoxic activity in presence of SM. Effect on cell viability of the combination of FC2 and SM83: (A) MDA-MB-231 and (B) SK-OV3 cells pretreated with different concentration of SM83, followed by addition of FC2 [0 (white bars), 12.5 (grey bars) and 100 μM (black bars)]. The experiments were repeated both in technical (three for each experiment) and biological (three independent experiments) triplicate. Mean values ± Standard deviation are shown.

For this reason, we tested the effect of the combination of FC2 and SM83, a divalent SM able to interact simultaneously with the BIR2 and BIR3 domains of IAPs [25]. FC2 enhanced the cytotoxic activity of SM83 in MDA-MB-231 and SK-OV3 cells (Fig. 4).

Experiments performed in MDA-MB-231 cells with serial dilutions of FC2 and of TNF (500 to 0.8 ng/ml) or SM83 (100 to 1.11 nM), revealed that the effects of FC2/TNF or FC2/SM83 combinations are additive [21].

Finally, we detected the fluorescence of a caspases 3/7 substrate added during cell viability assays on MDA-MB-231 cells, upon treatment with increasing concentrations of FC2. Since SMs are known to induce caspase activation [26], the positive control is represented by cells treated with SM83 (10 nM). Upon treatment with FC2, the activation of caspases 3/7 is detectable over the untreated control (Fig. S7, A), suggesting that FC2 induces a caspase-dependent cell death. A dose-dependent effect is also appreciable observing the fluorescence signals (Fig. S7, B).

#### 3.3.2 FC2 effect on NF-kB

**Fig. 5:**
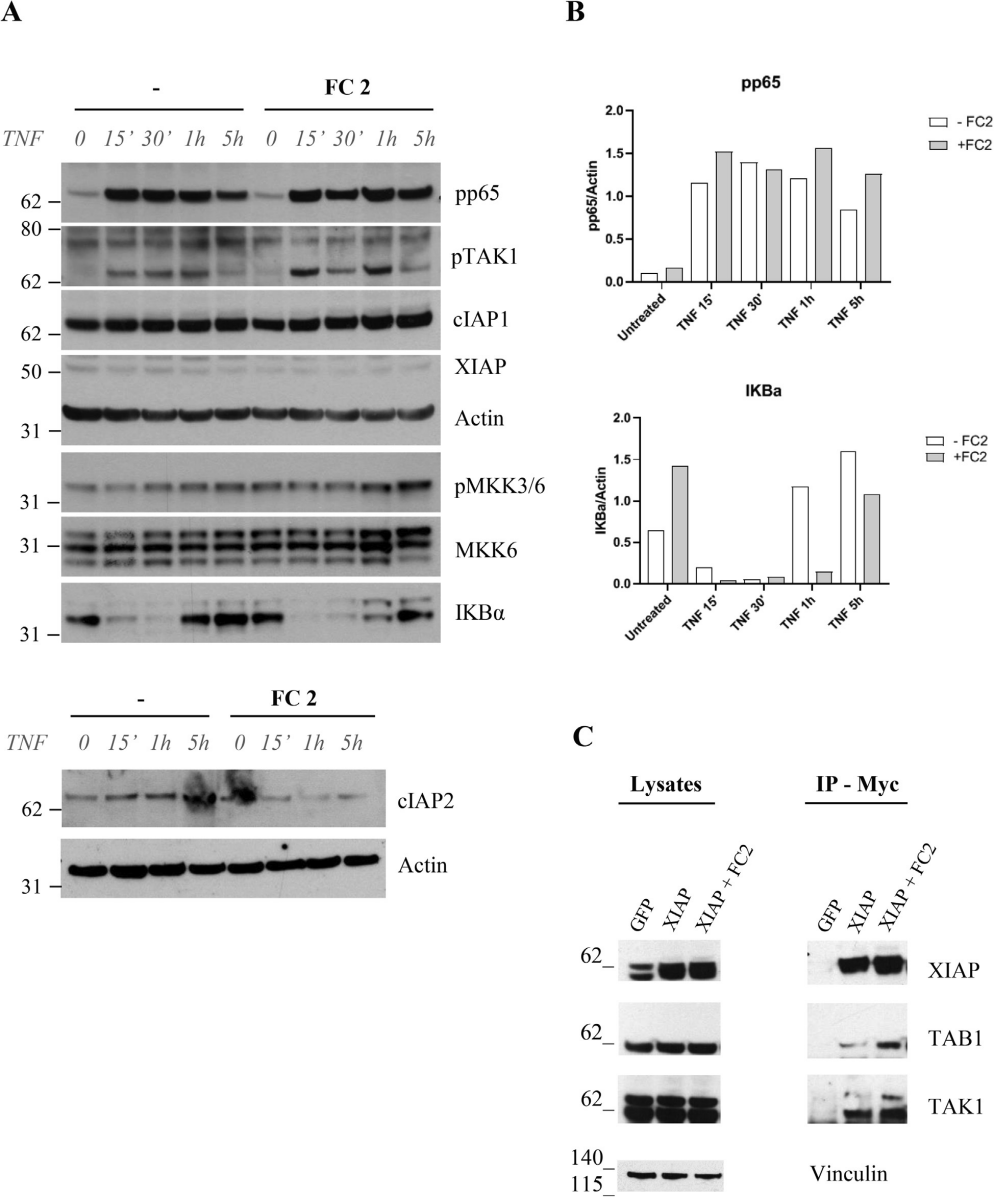
Effects of FC2 on the NF-κB pathway in MDA-MB-231 and HEK293 cells. A) MDA-MB-231 cells treated with 20 μM FC2 and then stimulated with 50 ng/mL TNF. NF-κB markers (pp65, pTAK1, cIAP1, cIAP2, XIAP, pMKK3/6, MKK6 and IκBα) were chosen to monitor the activation of the pathway. (B) In the presence of FC2, pp65 levels increase, suggesting a more prominent phosphorylation of p65 and NF- κB activation, and this effect is particularly evident at 1 and 5 h. Along these time ranges also IκBα levels are significantly different in the absence/presence of FC2, indicating a delay in protein levels’ recovery upon TNF-induced degradation. Densitometry analysis was performed with ImageQuant 5.2. C) Immunoprecipitation of XIAP overexpressed in HEK293. Left panel: lysates; right panel, immunoprecipitation of XIAP with Myc revealed a more pronounced recruitment of TAB1 and TAK1 upon treatment with FC2.

**Fig. 6:**
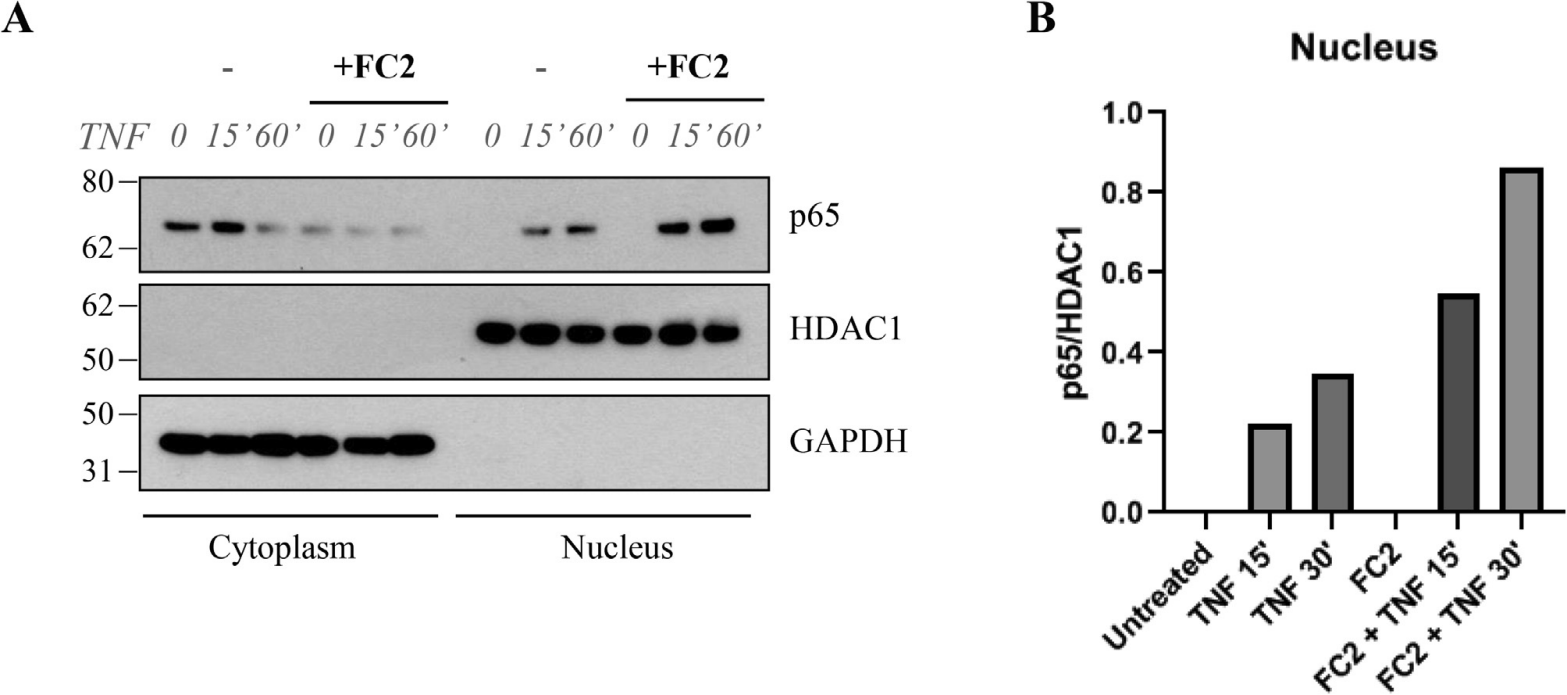
FC2 increases p65 translocation to the nucleus. A) MDA-MB-231 cells treated with 100 μM FC2 and then stimulated with 50 ng/mL TNF. Extraction of cytoplasmic and nuclear proteins revealed the decrease of p65 levels in the cytoplasm and an increase of p65 levels in the nucleus, suggesting a more pronounced activation of NF-κB pathway upon treatment with FC2. B) The effect of FC2 on p65 translocation to the nucleus is appreciable in the densitometry graph (ImageQuant 5.2), where the signal of p65 was normalized versus HDAC1.

To further investigate the mechanism of action of the compound, we analyzed the effects of FC2 treatment on NF-κB activation in MDA-MB-231 cells. TNF induced NF-κB activation is shown by the degradation of IκBα, whose levels recover after 1 h (Fig. 5, A). In contrast, a comparable recovery of IκBα can only be observed 5 h after FC2 administration. Accordingly, phosphorylation of p65, TAK1 and MKK3/6 is altered in the presence of FC2 after 5 h from TNF treatment (Fig. 5, A). A more pronounced activation of NF-kB pathway in the presence of FC2 was confirmed by detection of cytoplasmic/nuclear levels of p65 in MDA-MB-231 cells stimulated with TNF. As shown in Fig. 6, upon stimulation with TNF the decrease of p65 signal in the cytoplasm corresponds to an increase of the signal in the nuclear extract. This effect is more evident when cells are treated with FC2, suggesting a higher translocation of p65 in the nucleus, as a marker of NF-κB activation.

The molecular mechanism of XIAP-TAB1 mediated TAK1 activation is not fully understood [27]. We hypothesized that prolonged NF-κB activation could depend on the stabilization of the ternary complex XIAP-TAB1-TAK1 by FC2. As expected, FC2 treatment of HEK293 cells for 1 h, followed by XIAP immunoprecipitation (using Myc-tag), showed a slightly stronger interaction of the three proteins (Fig. 5, B).

## 4 Conclusions

BIR1 domains of IAPs are novel targets for drug discovery. Therefore in this work, we performed an *in silico* search for ligands of the BIR1 domains involved in the interaction with TAB1 and TRAF2, which allowed the identification of 20 best selected compounds. Among them, FC2 has been shown to induce cell death in human breast adenocarcinoma cell lines (MDA-MB-231, MDA-MB-468, HCC1419 and BT549) and ovarian tumor cells (SK-OV3), while showed no-toxicity effect in primary human fibroblast, suggesting a good safety profile (Fig. S1). We demonstrated that FC2 binds to the isolated BIR1 domains of XIAP and cIAP2 *in vitro* with low micromolar affinity and does not interfere with the dimerization of XIAP-BIR1 [12], [13] (Table S3). We tried to obtain a 3D structure of cIAP2-BIR1 bound to FC2 (Supplemental Fig. S3), however the diffracting co-crystal did not show any residual electron density attributable to the ligand, even if the crystallographic dimeric assembly of the protein (stabilized by a symmetric Cys28-Cys28 disulfide bridge) did not hinder the predicted binding site.

FC2 induces cancer cell death, and its activity increases with the addition of recombinant TNF (in MDA-MB-231 and SK-OV3) and is not reduced after suppression of autocrine TNF. The cytotoxic effect of FC2/TNF combination in MDA-MB-231 cells was demonstrated to be additive. Since TNF sensitivity arises from a plethora of cell-intrinsic mechanisms and depends on many pathways, different cell lines can display completely different responses to TNF stimulation ranging from apoptosis, necroptosis to increased aggressiveness. Accordingly, we found that, despite the combination with FC2, cell lines displayed different sensitivity to TNF administration (Fig. S2).

Interestingly, the combination of FC2 and SM83, a known IAP-antagonist [16], showed a cytotoxic effect that resulted additive. This evidence could be particularly useful in some cellular contexts where known IAP-antagonists show reduced efficacy [5].

The mechanism of FC2-induced cell death appears to be related to the prolonged activation of NF-κB, as indicated by the sustained degradation of IκBα. Another evidence supporting such hypothesis is the increased p65 translocation to the nucleus (Fig. 6). Since this evidence correlated with an increased phosphorylation of different protein targets, such as TAK1, we hypothesized that FC2 activity could be based on the stabilization of the XIAP/TAB/TAK complex. Accordingly, XIAP immunoprecipitation showed the FC2-induced stabilization of XIAP/TAB complex, which could enhance TAK1 phosphorylation, downstream p65 translocation to the nucleus and NF-κB activation.

Commercially available FC2 structural analogs were included in cell toxicity assays, providing a preliminary rationale for chemical optimization to start the design of a FC2-based library.

All the data collected suggest that FC2 represents the first candidate capable of inducing cancer cell death by interaction with type I BIR domains, proving to be an optimizable hit-to-lead molecule for the development of a new class of compounds able to tune NF-κB and BIR1 activities within pro-survival macromolecular complexes.

## 5 Databases

RCSB – Protein Data Bank ID 7NK0: Structure of the BIR1 domain of cIAP2.

## CRediT authorship contribution statement

**Federica Cossu:** Investigation, Software, Visualization, Writing – original draft. **Simone Camelliti:** Investigation, Software, Visualization, Writing – original draft. **Daniele Lecis:** Investigation, Methodology, Formal analysis. **Luca Sorrentino:** Investigation, Software, Visualization, Writing – original draft. **Maria Teresa Majorini:** Software, Formal analysis, Writing – review & editing. **Mario Milani:** Software, Supervision, Formal Analysis, Writing- original draft. **Eloise Mastrangelo:** Conceptualization, Funding acquisition, Writing – review & editing.

## Declaration of Competing Interest

The authors declare that they have no known competing financial interests or personal relationships that could have appeared to influence the work reported in this paper.
