# Supplementary material for: Structure-based identification of a new IAP-targeting compound that induces cancer cell death inducing NF-κB pathway
Source: Comput Struct Biotechnol J. 2021 Nov 26;19:6366–74. doi: 10.1016/j.csbj.2021.11.034 (PMC8649670; doi:10.1016/j.csbj.2021.11.034)
Supplement: Supplementary data 1 [file mmc1.docx]

**Supplementary Material**

**Material and Methods**

*S.1 Analytical Size Exclusion Chromatography*

To monitor X-BIR1 monomer/dimer equilibrium in the absence/presence of 2.5 mM FC2, analytical gel filtration runs were performed at different protein concentrations. The protein was progressively concentrated to a maximum concentration of 846 μM using an Amicon Ultra centrifugal filter (3 kDa cut-off). During size exclusion chromatography experiments sample volumes of 50 μl, with XIAP-BIR1 concentrations ranging from 45 μM to 846 μM, were injected on a Superdex 75 10/300 increase column (GE Healthcare ®) attached to an AKTA Pure 25 system in Tris-HCl, (pH 7.5, 20 mM), NaCl (200 mM) and DTT (10 mM). The dilution of all injected samples was estimated approximately 1:500. Low molecular weight standards from GE Healthcare ® were used to calibrate the column.

*S.2 Crystallization of cIAP2-BIR1*

Sitting drop crystallization experiments were prepared using an Oryx-8 crystallization robot (Douglas Instruments, East Garston, UK), from a 2:1 mixture of the protein stock solution (eventually provided with 2 mM of FC2) and the precipitant solution to a final drop volume of 0.3 µl for the initial screenings and of 0.5 µl for the optimization trials. After 4 days of vapor diffusion at 20°C, prismatic crystals of cIAP2-BIR1/FC2 were obtained in 20% (w/v) PEG 4000, 0.1 M Tris-HCl pH 8.5 and 0.2 M Lithium Sulfate. After soaking in paraffin oil and flesh-cooled in liquid nitrogen one crystal was used for data collection at the ESRF synchrotron in Grenoble (ID29). The diffraction experiment allowed to collect datasets at a maximum resolution of 3.3 Å.

*S.3 Structure determination and refinement*

The diffraction data were processed and scaled with XDS package (1). The cIAP2-BIR1 crystals belong both to the P6_4_ space group with unit cell parameters a = b= 93.3, c = 42.4 Å; there are two protein molecules in the crystal asymmetric unit (43% solvent content), and a disulfide bridge links the Cysteine residues in position 28 of two facing cIAP2-BIR1 molecules. The crystal structure was solved with molecular replacement (molrep (2)), using the BIR1 chain D in the TRAF2/cIAP2-BIR1 (PDB code 3M0A (3)) as search model. The two independent molecules were subjected to rigid-body refinement (R/Rfree = 29.5/35.6), and subsequently refined using REFMAC5 (4). A random set comprising 5% of the data was omitted from refinement for R-free calculation. Manual rebuilding (5) and additional refinement were subsequently performed (final resolution 3.3 Å).

The refined cIAP2-BIR1 structure displays 74 residues out of the 98 residues that compose our construct; the first 20 N-terminal residues (belonging to the expression vector) are disordered. Atomic coordinates and structure factors for cIAP2-BIR1 have been deposited with the Protein Data Bank (6) with accession code 7NK0.

**Results**

**Table S1.** *Citotoxic activity on MDA-MB-231 cells of candidate compounds resulting from virtual docking screening.* Inhibition constants predicted in virtual docking are reported for the best pose of each compound. Compounds FC1_1, FC4_1, FC6_1 (ChemBridge ID 6955650, 5216161 and 9288906, respectively) were purchased as structural water-soluble analogs of FC1, 4 and 6 (ChemBridge ID 7509055, 7630374 and 7463435), respectively. Each compound was added in the cell culture at different concentrations (100, 10, 1, 0 μM) in the absence/presence of TNF [50 ng/ml]. Notably, FC2 predicted Ki *vs* XIAP dimerization surface (2.7 μM) is comparable to FC2 predicted Ki *vs* XIAP surface interacting with TAB1 (1.0 μM). The percentage of survival (in black) and standard deviations (in orange) are calculated over three independent experiments.

| *Cmp* | **Pred. Ki [μM]**  **cIAP2**  **/**  **XIAP** | | **% Survival (Standard Deviation)** | | | | | | | | | | | | | | | |
| --- | --- | --- | --- | --- | --- | --- | --- | --- | --- | --- | --- | --- | --- | --- | --- | --- | --- | --- |
|  |  |  | **- TNF** | | | | | | | | **+ TNF 50 ng/ml** | | | | | | | |
|  |  |  | **100 μM** | | **10 μM** | | **1 μM** | | **0** | | **100 μM** | | **10 μM** | | **1 μM** | | **0** | |
| *FC1* | 0.3 | 2.6 | 78.2 | 31.6 | 104.7 | 27.8 | 101.6 | 13.1 | 100.0 | 0.0 | 71.5 | 7.9 | 86.3 | 3.5 | 92.8 | 3.0 | 100.0 | 0.0 |
| *FC1_1* |  |  | 93.8 | 14.2 | 118.9 | 38.1 | 130.5 | 55.6 | 142.8 | 37.9 | 85.1 | 19.0 | 114.9 | 14.6 | 120.5 | 25.5 | 120.9 | 17.3 |
| *FC2* | 1.0 | 2.7 | 16.8 | 3.7 | 72.6 | 35.0 | 77.5 | 5.6 | 116.6 | 19.4 | 17.6 | 4.8 | 34.6 | 12.4 | 72.0 | 1.2 | 105.0 | 16.5 |
| *FC3* | 3.8 | 1.3 | 23.5 | 1.3 | 108.0 | 36.2 | 91.3 | 1.0 | 142.5 | 40.9 | 31.2 | 12.2 | 128.0 | 23.6 | 107.7 | 13.9 | 119.9 | 15.3 |
| *FC4* | 6.5 | 1.8 | 55.4 | 16.7 | 90.4 | 9.2 | 99.7 | 5.4 | 131.5 | 32.9 | 43.6 | 14.5 | 96.8 | 1.1 | 111.3 | 10.4 | 116.6 | 30.1 |
| *FC4_1* |  |  | 25.2 | 27.5 | 122.2 | 52.1 | 129.8 | 48.0 | 131.1 | 66.2 | 26.5 | 10.3 | 110.0 | 13.7 | 87.5 | 14.0 | 99.5 | 21.8 |
| *FC5* |  | 3.5 | 117.2 | 26.6 | 132.5 | 40.7 | 122.3 | 17.5 | 132.1 | 31.7 | 49.8 | 25.4 | 103.4 | 8.6 | 119.9 | 13.0 | 118.1 | 13.5 |
| *FC6* | 5.0 | 3.9 | 120.2 | 18.8 | 141.7 | 35.8 | 133.6 | 20.4 | 147.5 | 53.0 | 97.8 | 20.3 | 115.9 | 27.4 | 130.0 | 35.0 | 130.1 | 34.3 |
| *FC6_1* |  |  | 87.1 | 15.7 | 107.7 | 18.9 | 126.1 | 38.4 | 139.0 | 55.6 | 85.0 | 2.0 | 101.5 | 12.9 | 113.9 | 10.1 | 114.6 | 2.1 |
| *FC7* |  | 1.3 | 47.1 | 30.4 | 114.7 | 19.1 | 111.9 | 7.0 | 140.6 | 46.5 | 22.9 | 3.6 | 107.9 | 1.0 | 110.6 | 12.9 | 118.8 | 16.0 |
| *FC8* |  | 0.3 | 70.9 | 30.8 | 71.0 | 29.3 | 76.8 | 22.6 | 80.7 | 18.0 | 70.7 | 30.0 | 74.5 | 23.0 | 81.6 | 16.3 | 86.8 | 8.9 |
| *FC9* | 4.2 | 2.0 | 77.1 | 28.0 | 84.6 | 13.3 | 93.5 | 7.2 | 88.8 | 9.6 | 57.7 | 30.7 | 86.1 | 15.6 | 88.8 | 5.9 | 81.5 | 16.5 |
| *FC10* |  | 1.4 | 80.2 | 13.5 | 91.6 | 4.2 | 94.6 | 4.0 | 96.6 | 0.1 | 79.9 | 3.0 | 98.1 | 0.3 | 94.7 | 0.1 | 94.7 | 2.0 |
| *FC11* |  | 1.7 | 4.6 | 1.0 | 67.4 | 24.5 | 92.1 | 1.9 | 102.4 | 9.5 | 12.7 | 7.7 | 92.2 | 1.6 | 98.2 | 3.8 | 94.2 | 3.2 |
| *FC12* |  | 1.8 | 83.0 | 16.2 | 96.8 | 3.4 | 89.4 | 16.3 | 100.7 | 1.6 | 62.1 | 39.9 | 94.6 | 7.2 | 88.5 | 14.2 | 99.7 | 4.6 |
| *FC13* |  | 2.7 | 96.6 | 1.9 | 99.8 | 1.1 | 97.4 | 4.8 | 99.1 | 4.0 | 84.9 | 23.9 | 105.9 | 3.9 | 87.8 | 14.1 | 108.1 | 13.1 |
| *FC14* |  | 3.4 | 31.3 | 38.7 | 102.9 | 9.9 | 99.3 | 7.0 | 102.9 | 4.4 | 25.6 | 27.1 | 106.7 | 11.6 | 103.4 | 11.3 | 101.0 | 2.6 |
| *FC15* |  | 2.6 | 58.6 | 30.6 | 81.4 | 33.5 | 78.0 | 33.3 | 106.8 | 1.5 | 61.2 | 54.5 | 66.2 | 54.4 | 94.5 | 13.5 | 107.2 | 1.2 |
| *FC16* |  | 3.6 | 76.1 | 29.2 | 106.3 | 1.6 | 102.2 | 6.1 | 108.3 | 2.5 | 92.0 | 10.9 | 109.3 | 1.3 | 106.5 | 5.1 | 117.5 | 12.3 |
| *FC17* |  | 3.1 | 90.3 | 17.2 | 104.9 | 9.6 | 93.4 | 13.6 | 113.8 | 10.5 | 87.7 | 10.8 | 117.6 | 22.9 | 114.6 | 23.9 | 109.3 | 2.0 |

**Table S2.** *Estimated inhibition constants (K_i_) and free binding energy values (ΔG) observed in virtual docking for FC2 and analogs are reported for cIAP2- and XIAP-BIR1 in the first two rows. The third row shows melting temperatures shifts of XIAP- and cIAP2-BIR1 [80 μM] in the presence of FC2 and FC2 analogs to a final concentration of 1 mM in 1% DMSO.* DMSO induces a slight variation of the proteins’ melting temperatures (cIAP2- and XIAP-BIR1 T_M_: 66.3 ± 0.2 and 62.0 ± 0.1. respectively; + 1% DMSO 66.4 ± 0.3 and 61.3 ± 0.06. respectively). Precipitates are observed upon addition of ligands, in variable extent. The melting temperature for each protein or protein-ligand adduct is calculated as the average of the lowest derivative values over triplicates. ΔT_M_ are calculated subtracting the average melting temperatures of protein-ligands adducts to the average values observed for the corresponding proteins with DMSO. Overall, all ligands induce an alteration of the melting curves of both proteins, displaying slowly increasing RFU (relative fluorescence units) values at low temperatures (20°-50 °C), before the temperature range where the inflection point is observed (50-70 °C). This indicates early unfolding events occurring at increasing temperatures in the range (20-50 °C). Such effect is particularly evident for analogs 1, 7 and 8. Interestingly, the melting curves in the presence of some compounds (as compounds 7 and 10 with cIAP2-BIR1, FC3 with XIAP-BIR1, and compound 1 for both protein constructs) display a biphasic shape, suggesting two different unfolding events. In these cases, two ΔT_M_ are reported in the table.

| ***cIAP2***  ***XIAP*** | ***1*** | ***FC2*** | ***FC3*** | ***4*** | ***5*** | ***6*** | ***7*** | ***8*** | ***FC9*** | ***10*** |
| --- | --- | --- | --- | --- | --- | --- | --- | --- | --- | --- |
| K_i_ (µM) | 17.4  1.7 | 1.0  0.25 | 3.8  2.6 | 10.1  3.5 | 5.0  0.5 | 2.4  1.3 | 6.1  4.9 | 3.6  1.1 | 4.2  0.8 | 4.3  4.2 |
| ΔG  (kcal/mol) | -6.49  -7.86 | -8.26  -8.98 | -7.40  -7.62 | -6.81  -7.44 | -7.23  -8.59 | -7.67  -8.01 | -7.12  -7.25 | -7.42  -8.14 | -7.34  -8.29 | -7.32  -7.33 |
| ΔT_M_ | -29.4  +2.3  -22.8  +0.6 | -3.0  -1.5 | -9.0  -3.5  -17.3 | +0.6  -0.8 | -2.1  -1.8 | -0.5  +2.8 | -16.4  -1.4  -1.5 | +1.3  -1.3 | -1.5  -0.4 | +18.4  +0.4  +21.3 |

**Table S3.** *Size exclusion chromatography on increasing concentrations of XIAP-BIR1 in the absence/presence of 2 mM FC2.* XIAP-BIR1 is known to elute as an equilibrium between its monomeric and dimeric form (XIAP-BIR1 theoretic molecular weight 11.03 kDa). The addition of FC2 does not interfere with the monomer/dimer equilibrium, which was monitored at increasing protein concentrations, ranging from 45 to 846 μM.

| [X-BIR1] (μM) | V_E_ (-,+FC2) | Estimated Mw (kDa) |
| --- | --- | --- |
| 45 | 14.04, 14.03 | 13.11, 13.16 |
| 90 | 13.95, 13.98 | 13.58, 13.42 |
| 170 | 13.83, 13.84 | 14.22, 14.17 |
| 680 | 13.48, 13.50 | 16.28, 16.15 |
| 846 | 13.37, 13.44 | 16.99, 16.53 |

**Figure S1.** *Evaluation of FC2 toxicity as single agent on primary human fibroblasts and on additiona human breast adenocarcinoma cell lines.* **A)** FC2 is not toxic in BJ cell line (human primary fibroblast). **B, C)** FC2 was tested also on additional breast adenocarcinoma cell lines, MDA-MB-468 (triple negative breast adenocarcinoma) and HCC1419 (HER2+ breast adenocarcinoma). In the first cell line, FC2 toxicity was comparable to the effect observed on MDA-MB-231. In HCC1419 cell line, FC2 effect is similar to the toxicity observed in BT549 cell line.

**A**

**B**

**C**

**Figure S2.** *FC2 induces cell death also in BT549* ***(A)*** *and SK-OV3* ***(B)*** *cells, displaying a potentiated activity in the presence of TNF in SK-OV3.*

**B**

**A**

**Figure S3.** *Crystal structure of cIAP2-BIR1.* The structure of cIAP2-BIR1 (PDB ID: 7NK0) reveals a crystallographic dimer stabilized by a disulphide bridge between two facing α1 helices.


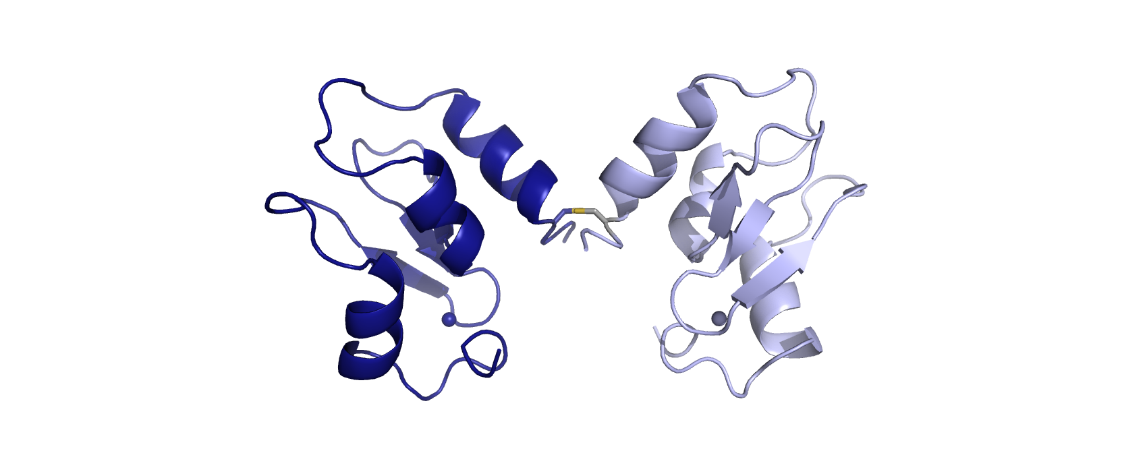


cIAP2-BIR1 (E)

cIAP2-BIR1 (D)

Cys28-SS bridge

**α1**

**α2**

**α3**

**β1**

**β2**

**β3**

**Zn^2+^**

RCSB ID: 7NK0

R = 3.3 Å

Rfac/Rfree = 0.24/0.33

N-ter

C-ter

C-ter

**Figure S4.** *FC2 is predicted to interfere with cIAP2/TRAF2 and XIAP/TAB1 interactions.* The predicted pose of FC2 (green sticks) targets a structural hotspot on cIAP2-BIR1 (light blue cartoon, **A**) and XIAP-BIR1 (light green cartoon, **B**) involving the C-terminal part of α1 and the α1- α2 loop, a region shown to engage contacts with TRAF2 (pink cartoon/surface, PDB code 3M0A, 10) and TAB1 (purple cartoon/surface, PDB code 2POP, 8).

**B**

**A**


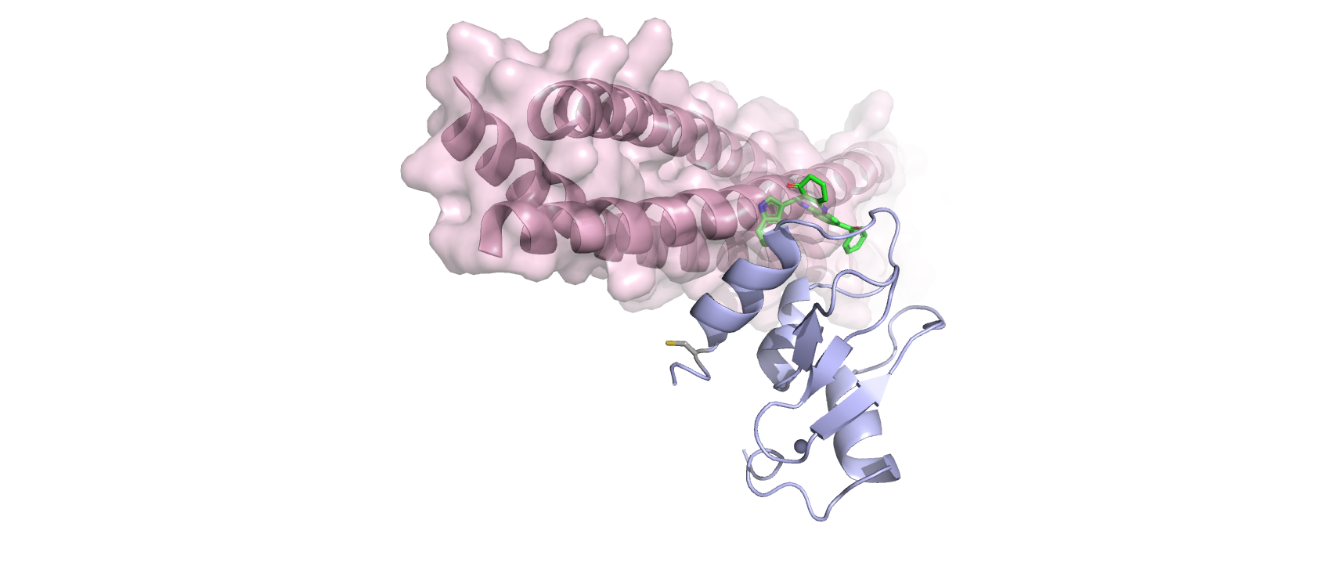


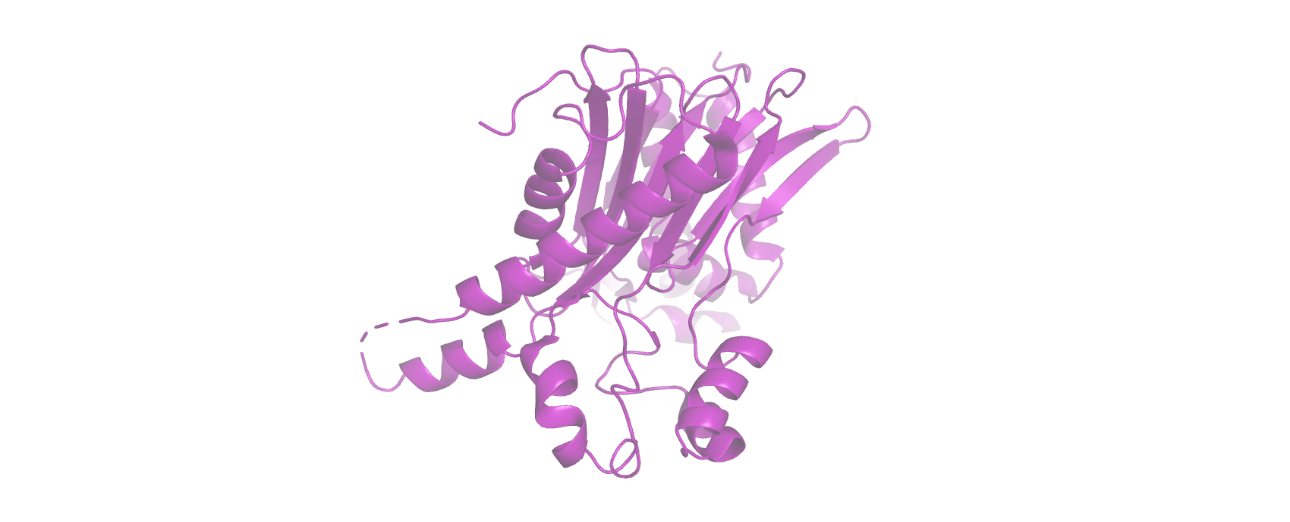

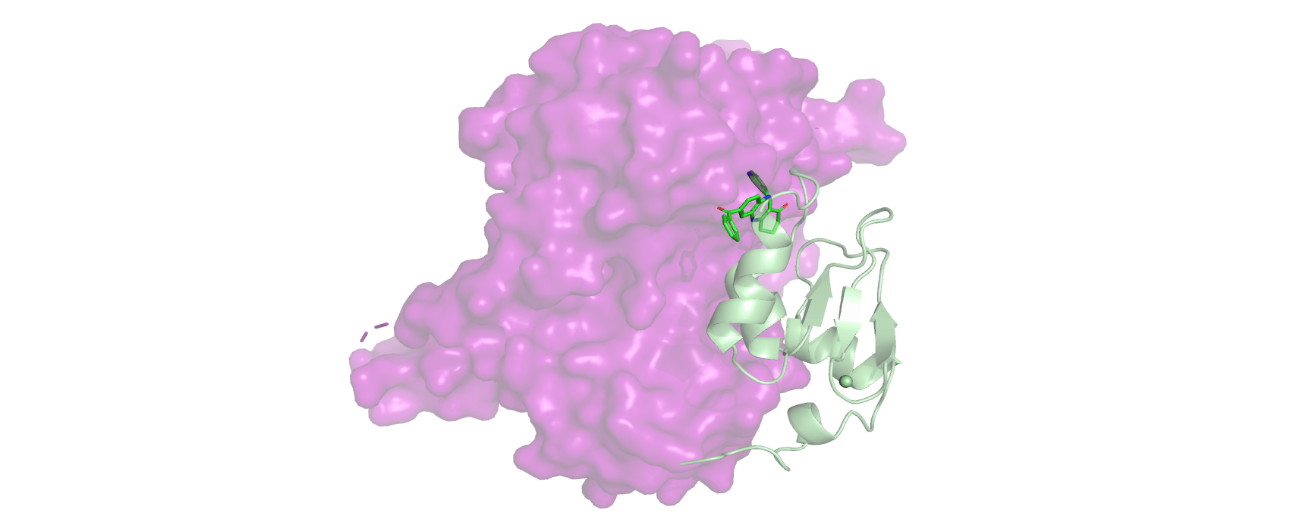


TRAF2

**FC2**

**FC2**

TAB1

Cys28

cIAP2-BIR1

XIAP-BIR1

**Figure S5.** *NMR data of FC2 compound provided by Chembridge Corp*.

**

**

**Figure S6.** Light microscope observation of FC2 toxicity on MDA-MB-231 cells at different FC2 concentrations (A: 25, B: 12.5, C: 6.25, D: 0 μM).


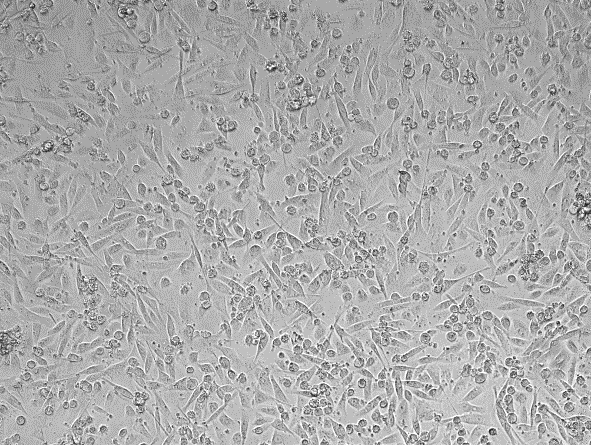

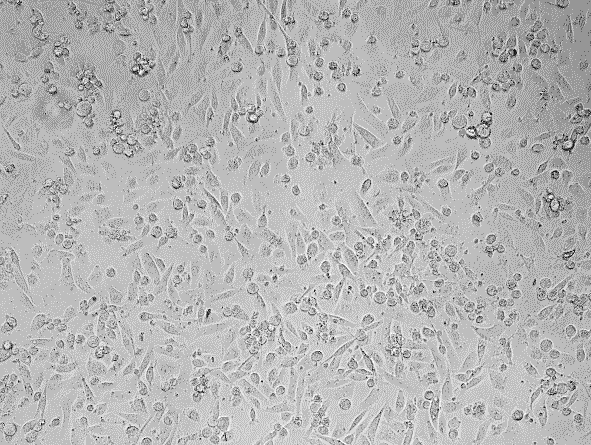

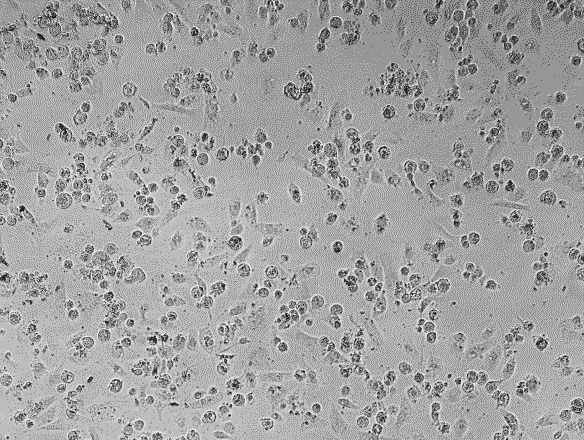

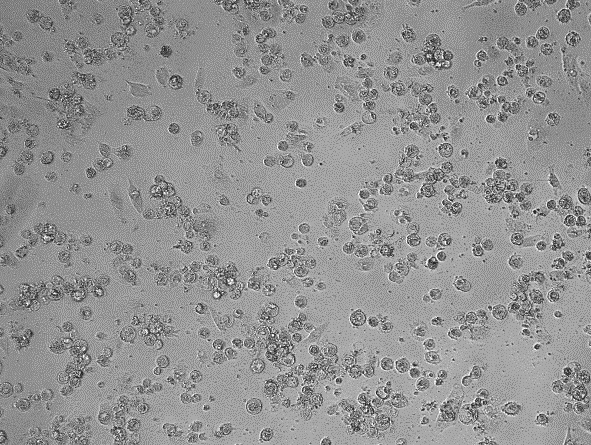


**A**

**B**

**C**

**D**

**Figure S7.** *Caspases 3/7 activation upon FC2 treatment.* MDA-MB-231 cells were treated with increasing concentrations of FC2 (100 to 3.125 μM). Untreated wells and wells added with 10 nM SM83 were left for negative and positive controls, respectively. The fluorescence signals reveal activation of effector caspases in wells treated with SM83 and FC2 (**A**, zoom 10x). The increase of fluorescence detected is appreciable upon increasing FC2 concentrations (**B**, zoom 20x). All experiments were performed in technical triplicate.

**A**


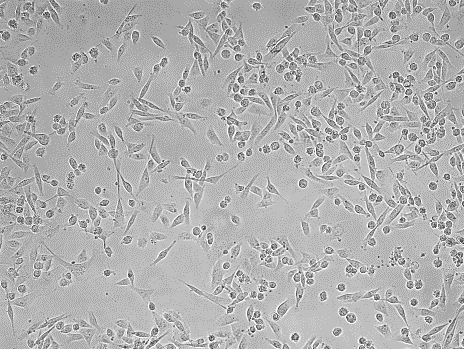

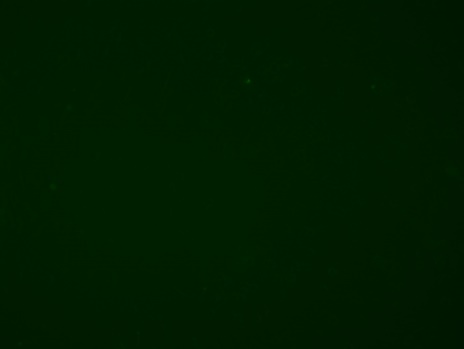

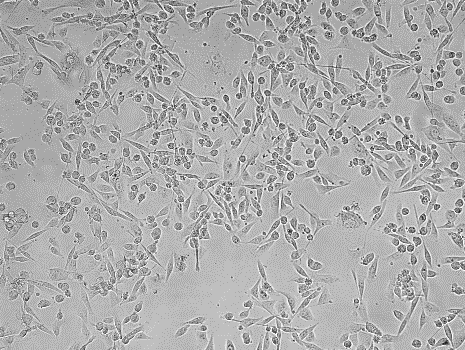

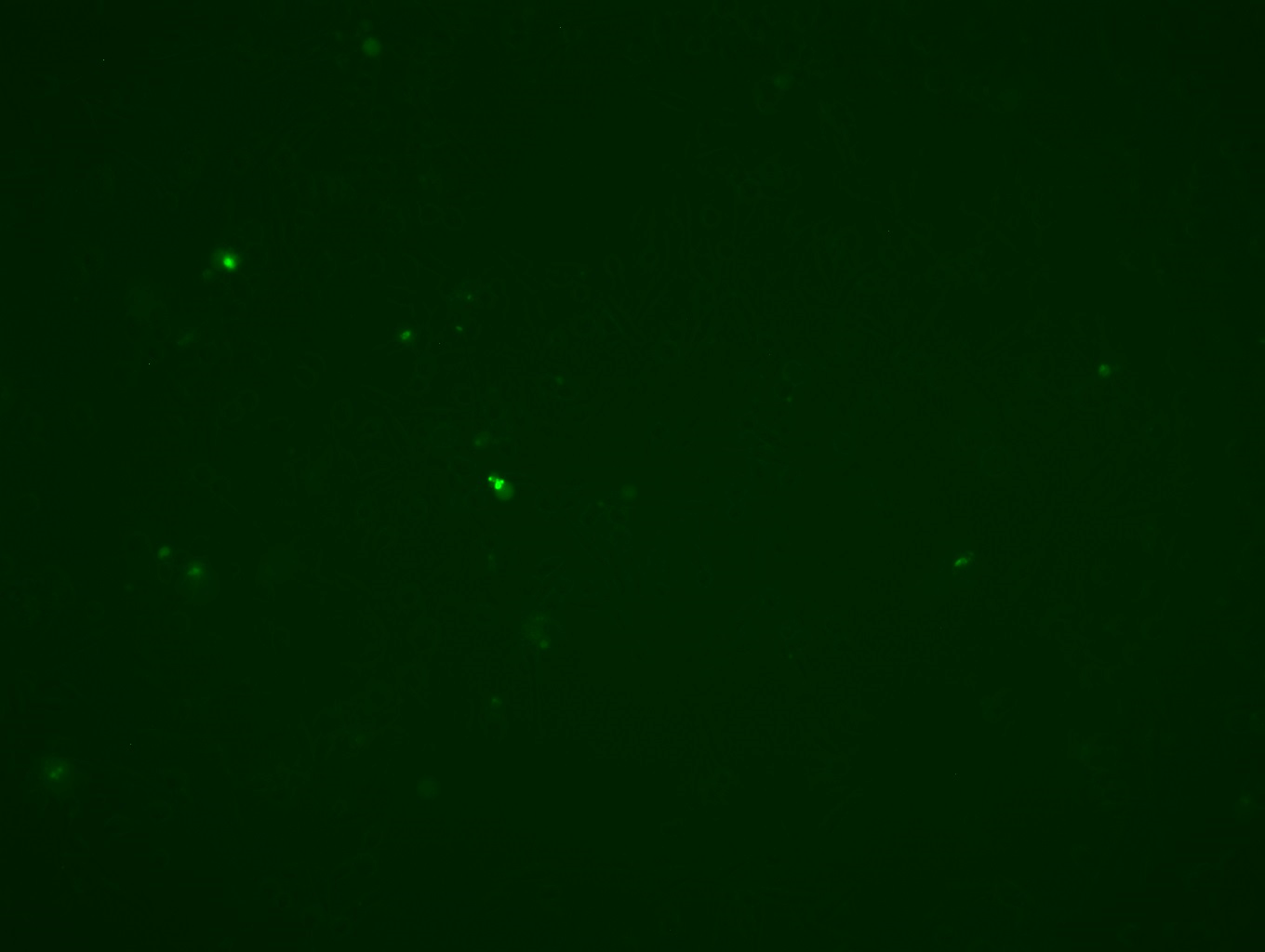


**10x**

**10x**

**10x**

SM83, 10 nM

48 h – FC2, 50 μM

Untreated


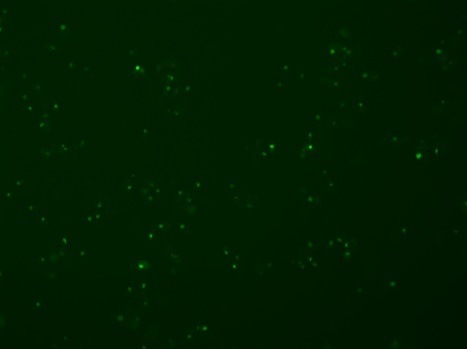

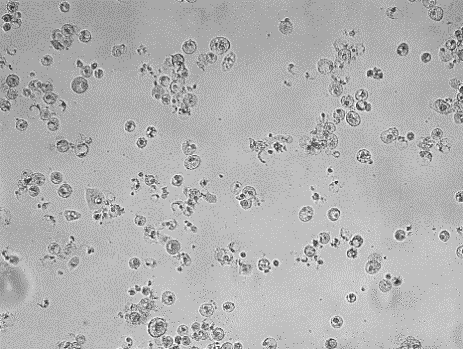


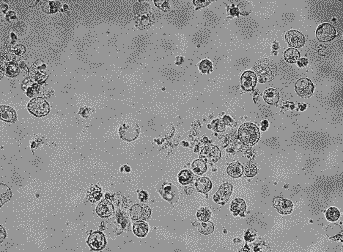

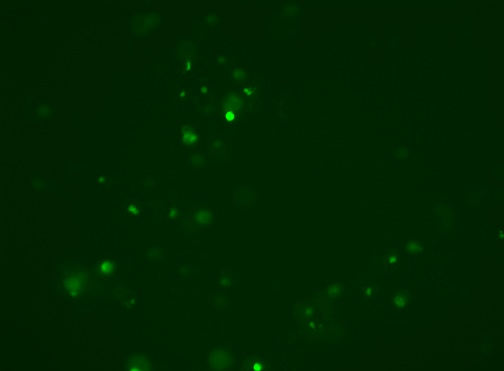

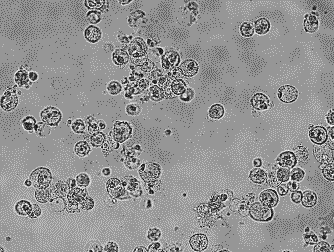

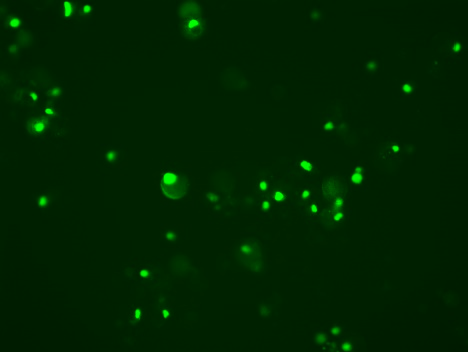
**B**

48 h – FC2, 50 μM

48 h – FC2, 25 μM


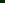

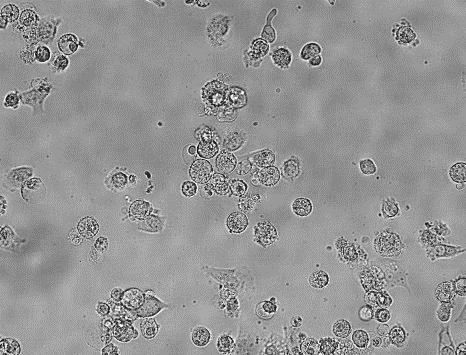

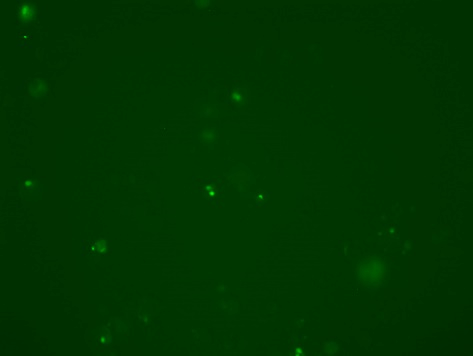


**20x**

48 h – FC2, 12.5 μM

**20x**

**20x**


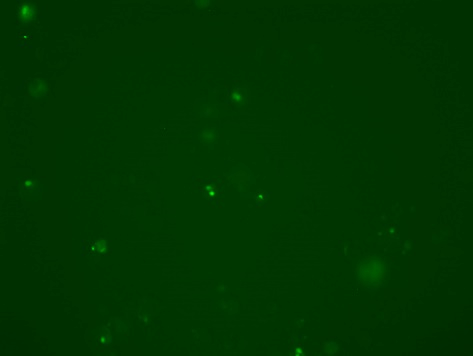

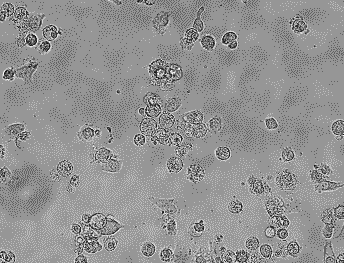


48 h – FC2, 12.5 μM

**20x**

**Supplementary References**

1. Kabsch W. Automatic processing of rotation diffraction data from crystals of initially unknown symmetry land cell constants. J Appl Crystallogr. 1993;

2. Vagin A, Teplyakov A. *MOLREP* : an Automated Program for Molecular Replacement. J Appl Crystallogr. 1997;30:1022–5.

3. Zheng C, Kabaleeswaran V, Wang Y, Cheng G, Wu H. Crystal Structures of the TRAF2: cIAP2 and the TRAF1: TRAF2: cIAP2 Complexes: Affinity, Specificity, and Regulation. Mol Cell. 2010;

4. Winn MD, Isupov MN, Murshudov GN. Use of TLS parameters to model anisotropic displacements in macromolecular refinement. Acta Crystallogr Sect D Biol Crystallogr. 2001;57:122–33.

5. Emsley P, Cowtan K. Coot: Model-building tools for molecular graphics. Acta Crystallogr Sect D Biol Crystallogr. 2004;60:2126–32.

6. Berman HM, Westbrook J, Feng Z, Gilliland G, Bhat TN, Weissig H, et al. The protein data bank. Nucleic Acids Res. 2000;28:235–42.
